# Resolutions of Respect—Dr. V. H. Taliaferro

**Published:** 1887-10

**Authors:** Hunter P. Cooper, James B. Baird, J. S. Todd

**Affiliations:** Committee; Committee; Committee


### RESOLUTIONS OF RESPECT.

### THE ATLANTA SOCIETY OF MEDICINE MEETS AND PAYS RESPECT TO DR. TALIAFERRO'S MEMORY.

The Atlanta Society of Medicine met in regular monthly session yesterday afternoon in the hall on Broad street. There was a large attendance, and the meeting was presided over by Dr. Hardon, the President.

During the meeting the President appointed a committee of three to draft resolutions touching the death of Dr. Taliaferro. The committee was composed of Dr. Hunter P. Cooper, Dr. James B. Baird and Dr. J. S. Todd. The gentlemen retired, and in a short time returned with the following resolutions of respect, which were adopted:

WHEREAS, God, in His inscrutable wisdom, has removed from this life our beloved colleague, Dr. VALENTINE H. TALIAFERRO—

*Resolved*, That while we deeply mourn our loss, we bow in submission to the decrees of an all-wise Providence.

*Resolved*, That the death of Dr. Taliaferro is an irreparable calamity, not only to our Society, but to the medical profession at large.

*Resolved*, That we extend to his family our deepest sympathy in their terrible affliction.

*Resolved*, That these resolutions be published in *The Atlanta Constitution* and in THE ATLANTA MEDICAL AND SURGICAL JOURNAL.

HUNTER P. COOPER, M. D.,
JAMES B. BAIRD, M. D.,
J. S. TODD, M. D.,
Committee.

